# The development of sensitive graphene-based surface acoustic wave sensors for NO_2_ detection at room temperature

**DOI:** 10.1007/s00604-024-06397-y

**Published:** 2024-05-10

**Authors:** Valentin Buiculescu, Livia Alexandra Dinu, Lucia Monica Veca, Cătălin Pârvulescu, Madalina Mihai, Oana Brîncoveanu, Florin Comănescu, Costin Brașoveanu, Marius Stoian, Angela Mihaela Baracu

**Affiliations:** National Institute for Research and Development in Microtechnologies (IMT Bucharest), 126A Erou Iancu Nicolae Street, 077190 Voluntari (Ilfov), Romania

**Keywords:** SAW sensors, Sulphur-doped graphene, Bilayer graphene, NO_2_

## Abstract

Bilayer graphene (Bl-Gr) and sulphur-doped graphene (S-Gr) have been integrated with LiTaO_3 _surface acustic wave (SAW) sensors to enhance the performance of NO_2_ detection at room temperature. The sensitivity of the Bl-Gr SAW sensors toward NO_2_, measured at room temperature, was 0.29º/ppm, with a limit of detection of 0.068 ppm. The S-Gr SAW sensors showed 0.19º/ppm sensitivity and a limit of detection of 0.140 ppm. The origin of these high sensitivities was attributed to the mass loading and elastic effects of the graphene-based sensing materials, with surface changes caused by the absorption of the NO_2_ molecules on the sensing films. Although there are no significant differences regarding the sensitivity and detection limit of the two types of sensors, the measurements in the presence of interferent gases and various humidity conditions outlined much better selectivity and sensing performances towards NO_2_ gas for the Bl-Gr SAW sensors.

**Graphical Abstract:**

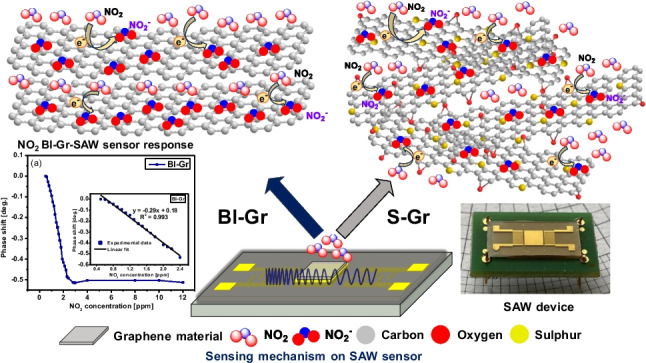

**Supplementary Information:**

The online version contains supplementary material available at 10.1007/s00604-024-06397-y.

## Introduction

Environmental issues represent one of the biggest concerns nowadays, hence, new ways to detect and monitor ambient air pollutants and toxic gases become critical. One of the most dangerous species is nitrogen dioxide (NO_2_), accountable for acid rains, and ozone formation, being a major environmental cause of morbidity and mortality worldwide, in case of repetitive or short-term exposure, even in low concentrations of 100 ppb (1-h average exposure, World Health Organization—WHO) [1–3]. The short-term exposure limit for NO_2_ is 1 ppm, a value recommended by many safety and health organizations, such as the European Chemicals Agency [4] the National Institute for Occupational Safety and Health (NIOSH), Occupational Safety and Health Administration (OSHA) [5], etc. Ensuring compliance with such limits requires individual and continuous NO_2_ gas monitoring.

The most used sensors for NO_2_ detection are based on chemiresistive devices and surface acoustic wave (SAW) structures. Despite the different gas sensing mechanisms, both types of sensors use NO_2_-sensitive materials. Over time, various promising sensing nanomaterials, including colloidal quantum dots [6–8], thin films of metal oxide semiconductor [9–14], or carbon-based nanomaterials [15–19] have been employed as gas-adsorption films, leading to enhanced sensitivity of the NO_2_ sensor. Amongst the mentioned sensing films, graphene has been the focus of intense research in recent years due to its unique electronic and mechanical properties, and high chemical stability [20].

Graphene is particularly attractive for NO_2_ detection due to its large specific surface area and room temperature (RT) operation ability. These features led to the development of NO_2_ sensors with extremely low limits of detection (LOD). For example, by using a NO_2_ chemiresistive sensor based on chemical vapor deposited (CVD) graphene, an extremely low LOD of 2.06 ppt was achieved. The respective LOD was attributed mainly to the minimal noise that is generated by the very low resistivity of CVD-graphene (10^−6^ Ω cm) [21]. However, most of the NO_2_ graphene-based chemiresistive sensors suffer from low selectivity and their sensing performances are highly affected by humidity and temperature when operating under ambient conditions, restricting their extensive usage [22].

These shortcomings can be overcome by using surface acoustic wave (SAW) sensors, which are another promising alternative method to detect de NO_2_ gas. The deployment of SAW devices for sensing applications is an emerging area of research due to their miniaturization, high sensitivity, operation without DC power sources, ability to be used for on-site detection, and fast response time along with inherent passive nature [23]. In addition, the fabrication process of SAW devices is fully compatible with microelectronics technology, which offers customized design, scalability, possibility of integration with micro-electromechanical systems (MEMS) [24–28] for smart sensors’ development and high throughput mass production. Moreover, room temperature NO_2_ detection can be easily achieved by integrating SAW devices with graphene-based materials. The sensing properties of graphene are highly dependent on the preparation methods and fabrication processes, which influence its physical morphology, surface chemistry, and electrical features. Therefore, researchers have focused on various methods to obtain, modify, and enhance the properties of graphene for NO_2_ sensing applications.

Bilayer graphene (Bl-Gr) deposited by CVD has received increasing attention for NO_2_ sensing due to its two-dimensional properties, similar to monolayer graphene, but with superior electrical quality with Bernal AB stacking order [29, 30]. Moreover, the structure of Bl-Gr presents more available active sites compared to monolayer graphene, leading to improved adsorption of the NO_2_ molecules.

Another promising approach to enhance the graphene affinity to NO_2_ is the doping heteroatom, which involves introducing atoms of different elements, such as sulphur, into the graphene lattice [31]. Heteroatom doping introduces defects into the graphene lattice and modifies its electronic structure, resulting in enhanced sensitivity to specific analytes [32]. In particular, sulphur-doped graphene (S-Gr) has shown enhanced sensitivity and selectivity for gas applications [33], while the large surface area makes it a promising material for SAW sensors.

This paper reports the development of NO_2_ SAW sensors based on both Bl-Gr and S-Gr nanomaterials and presents their comparison in terms of sensing performances, exploring the potential impact of this type of sensing system on the future of gas sensing technology.

## Materials and methods

### Reagents and instrumentation

Sulphur-doped graphene, potassium persulfate, and dimethyl formamide (DMF) were purchased from Sigma Aldrich (Germany), polymethyl methacrylate (950 PMMA A4), lift-off resist (LOR) 5A, AZ 1518 positive photoresist and AZ 726 developing solution were purchased from MicroChem. LiTaO_3_-36ºYX piezoelectric wafers were purchased from the Roditi International Corporation Ltd. Monolayer graphene on copper foil was purchased from Graphenea, with the following characteristics: CVD synthesis as growth method, 0.345 nm theoretical thickness, ≈1500 cm^2^/V·s electron mobility on SiO_2_/Si and 350 ± 40 Ω/sq sheet resistance on SiO_2_/Si (an area of 1 cm × 1 cm was considered).

The (photolithographic) masks writing process was performed by Pattern generator—DWL 66 fs Laser Lithography System (Heidelberg Instruments Mikrotechnik, Germany), the metallic thin films were deposited by the Electron Beam Evaporation System (Temescal FC-2000, USA), while the SiO_2_ guiding layer was deposited by the Plasma-Enhanced Chemical Vapor Deposition (PECVD—LPX-CVD, with LDS module, STS, UK). Electrical connections between open packages and sensor chips were performed by Au wires using the Wire Bonder System equipment (HB05, Nano Vacuum Pty Ltd, Australia). The morphological and compositional characterization of Gr-based nanomaterials was performed by the Nova NanoSEM 630 Scanning Electron Microscope (FEI Company, Hillsboro, OR, USA) using a UHR detector (Through-Lens-Detector-TLD) at an acceleration voltage of 10 kV and an element energy dispersive spectroscopy (EDS) system (Smart Insight AMETEK). The Raman spectra were carried out at RT (24 °C) using a LabRAM HR800 Raman spectrometer in setup with a He–Ne laser. All sensors’ measurements in the NO_2_ (target analyte), CH_2_O, NH_3_, and CO (interfering gases) atmosphere were carried out by the high-resolution vector network analyzer (VNA), Anritsu model 37397D.

### Design and fabrication of the surface acoustic wave devices

The SAW-delay line (DL) devices were fabricated by conventional photolithography technology on lithium tantalate (LiTaO_3_-36ºYX) piezoelectric substrate. This material allows the propagation of shear waves, with a phase velocity of 4150 m/s, and benefits from a very high coupling coefficient and moderate temperature dependence. The proposed SAW structures use a SiO_2_ guiding layer, providing very high stability to the sensors and high sensitivity to the surface perturbation due to the lower acoustic wave velocity in the guiding layer as compared to the wave velocity in the piezoelectric substrate. Moreover, the amorphous SiO_2_ layer also improves the temperature stability of the SAW device due to its positive temperature coefficients of elastic constants, opposite to those of the LiTaO_3_ piezoelectric substrate. The design parameters of the guided DL-SAW devices are listed in Table S1, resulting in an operating frequency of 121 MHz for the proposed sensors.

To reduce the cost of the entire SAW manufacturing process, a three-step technological flow was proposed (Figure S1). The fabrication process started with the cleaning of the LiTaO_3_ piezoelectric substrate in acetone, aimed to remove organic contaminants and impurities, followed by repeated rinsing in isopropyl alcohol and drying by centrifugation at 6000 rpm. The wafers were then dehydrated at 150 °C for 5 min on the hot plate.

The first step of the technological flow consisted of a bi-layer photoresist deposition to configure the IDTs. The first layer of LOR 5A was deposited by spin coating at 3000 rpm for 60 s, followed by a soft bake at 155^0^ C for 5 min. The second layer of AZ 1518 positive photoresist was spin-coated at 4000 rpm for 60 s and soft-baked at 95 °C for 45 s, resulting in a thickness of 1.8 µm. Exposure of the first photolithographic mask was performed at 405 nm irradiation for 2.8 s (39.2 mJ). The exposed photoresist was developed in AZ 726 developing solution for 50 s.

A thin layer of 15 nm of Cr was initially deposited to obtain good adhesion to the substrate, followed by the deposition of 150 nm Au film, using e-beam evaporation. The lift-off process was carried out by immersion of the wafers in hot acetone for 5 min. As the layer of LOR 5A was not dissolved in acetone, it was removed by immersion in a solution based on potassium hydroxide (KOH), with a concentration of 1.33%, for 1 min.

A 3 µm thick SiO_2_ guiding layer was deposited at 150 °C by PECVD from the SiH_4_ precursor, confining the acoustic energy near the surface and protecting the IDTs during the functionalization protocols of the SAW device, since the distance between the sensing area and IDTs is small (only 190 µm).

The second photolithographic process consisted of a new deposition and patterning of AZ 1518 positive photoresist to open the electric contacts (pads) in the guiding layer. Etching of SiO_2_ through the photoresist mask was performed in a buffered oxide etchant (BOE) solution based on a mixture of hydrofluoric acid (HF) and ammonium fluoride (NH_4_F) (v:v 6:1). To control the etching rate, the solution was kept at a temperature of 23 °C.

The third photolithographic process, consisted of the configuration of the detection area of the SAW device, followed the same photolithographic processes described in the first step of the technological flow.

**Fig. 1:**
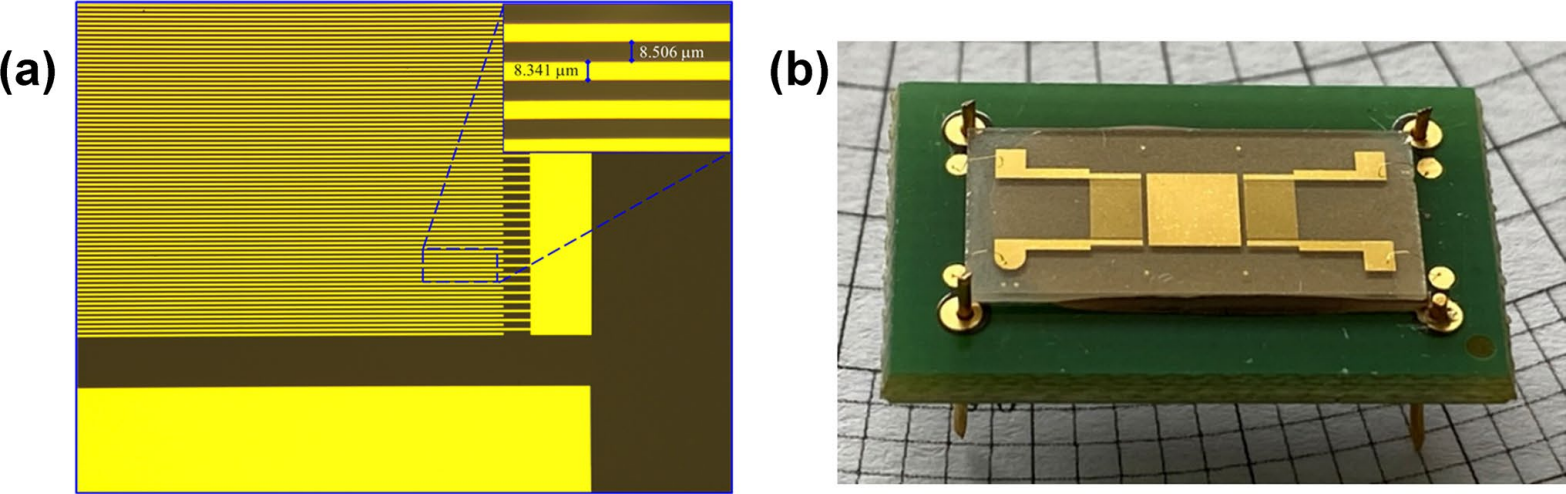
Image of the SAW structure on LiTaO_3_ piezoelectric substrate: (**a**) IDT details and (**b**) encapsulated SAW sensor

At the end of the technological processes, the structures were investigated using optical microscopy. The areas of interest were represented by the IDTs’ electrodes. Details of the structures obtained onto LiTaO_3_ substrate can be seen in Fig. 1(a). After the optical inspection, the wafers were diced into chips and prepared for encapsulation (Fig. 1(b)). Open packages (OPs) made on FR-4 substrate as printed circuit boards (PCBs) with two metal layers (Figure S2) were used for chips’ encapsulation.

### Design of the GR-based SAW sensors

The packaged SAW devices were further functionalized by using Bl-Gr and S-Gr sensing nanomaterials for the sensitive and selective detection of NO_2_.

#### The transfer protocol of the Bl-Gr

The transfer of the graphene (Gr) layer from the growth substrate to the SAW device employed an electrochemical delamination approach. To this end, the commercial monolayer graphene on copper foil (1.5 × 1.5 cm^2^) was first spin-coated with PMMA at 1100 rpm for 1 min and then cured on a hot plate at 150 °C for 2 min. Subsequently, a polyethylene terephthalate (PET) frame with an inner diameter of 4 mm was placed on top of the PMMA/Gr/Cu stack. Electrochemical delamination of the graphene monolayer was carried out in 0.05 mM aqueous solution of potassium persulfate (K_2_S_2_O_8_), with the PET/PMMA/Gr/Cu foil and graphite as cathode and anode of the electrolytic cell. The floating PET/PMMA/Gr stack was subsequently hand-picked, rinsed in deionized water several times, and placed on the predefined sensing area of the SAW device, releasing thereafter the PMMA/graphene from the PET frame. After 1 h at room temperature (24 °C), the PMMA layer was removed in acetone. Following the same procedure, the second graphene layer was placed on top of the first graphene layer, finishing with the PMMA removal in acetone.

#### Drop-casting of S-Gr dispersion

The preparation protocol, previously reported in [34], was based on dispersing the S-Gr in DMF, under ultrasonication for 10 min at 30 °C, in a final concentration of 1.33 mg/mL. The functionalization of the SAW devices is illustrated in Figure S3. The dispersed S-GR was drop-casted on top of the square sensing area of the SAW device, under thermal treatment, on a hotplate [35]. The dispersion was deposited at 1 µL at a time until a final volume of 10 µL was reached. In the end, the functionalized device was removed from the plate and let to cool down and dry completely for at least 12 h. The dispersion protocol takes no more than 5–7 min after the plate is heated. Since more than 15 devices can be functionalized at the same time, on the hot plate, this functionalization technique has proven to be very fast.

### Gas sensing measurements

Because of the NO_2_ toxicity, the sensors used to measure the concentration of this analyte were placed in a sealed stainless-steel enclosure, and provided with separate nozzles for the admission and exhaust of the gas mixtures. The enclosure accommodates up to two sensors for measurements under controlled atmosphere conditions. A removable stainless-steel lid seals the measuring enclosure using a rubber gasket inserted in a shallow groove.

Since the sensor was inserted in a sealed enclosure, without direct access to its ports, scattering matrix measurement requires the addition of interconnection elements specific to the high-frequency techniques: coaxial cables, adapters between different types of connectors, etc. Moreover, the sensor package was connected to these interconnection elements using a compatible socket. Therefore, hermetically sealed SMA jack to bulkhead jack adapters model SF2991-6002 from Amphenol SV Microwave, passing through the cavity lid, allowed the high-frequency signal to access the sensor ports to determine their response to the concentration of analytes introduced into the enclosure.

Accurate characterization of the NO_2_ sensors required calibration of the measurement system, which resulted in transferring the reference planes of the VNA to the terminals of the socket into which the packaged sensor was inserted. A calibration procedure was performed using short, open, load, through (SOLT) circuit elements [36] designed and manufactured as PCB, fully compatible with the layout of the assembly socket. Once the calibration protocol was completed, the measurement system was ready to determine the specific characteristics of NO_2_ sensors at 24 °C. In the case of sensors operating by signal transmission at high frequencies, sensitivity refers to the change in the measured forward transfer scattering parameter S_21_ (i.e., amplitude and/or phase) as a function of actual analyte concentration. Since the amplitude characteristic of the NO_2_ sensors changes very slowly over a wide frequency band, on top of which periodic fluctuations overlap [35], it was not considered suitable for sensitivity measurements. Consequently, only the phase variation of the transfer coefficient vs NO_2_ concentration was used for this operation.

**Fig. 2:**
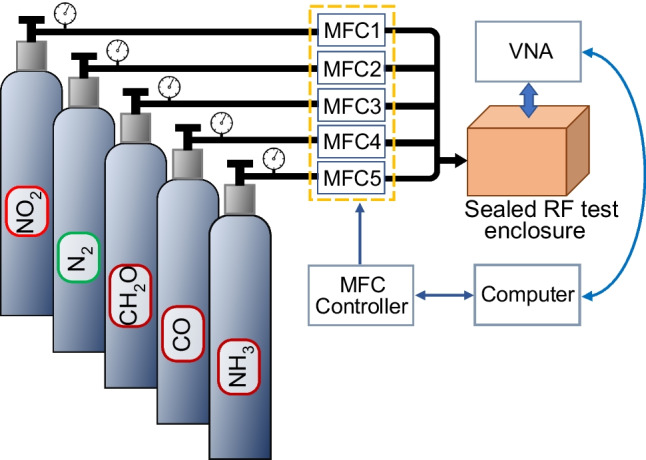
The experimental setup used for gas sensing tests of SAW sensors

The simplified representation of the gas sensing experimental setup is illustrated in Fig. 2. The fabricated sensor was placed into a sealed cavity, externally connected to a network analyzer and a PC. The gaseous analytes used for sensitivity and selectivity measurements were diluted with nitrogen (N_2_), and stored in pressurized cylinders. The gas mixture was injected into the sealed cavity containing the SAW sensor(s) under test at the rate of 500 sccm. This value was selected to ensure a laminar flow, otherwise, measurement errors may occur on the phase transfer coefficient due to temperature changes caused by compression or extension of the gas mixture at the inlet to the measurement enclosure. The temperature (24° C) and humidity in the sealed test cavity were maintained constant throughout the experiment.

## Results and discussions

### Morphological and structural characterization of Gr-based nanomaterials

**Fig. 3:**
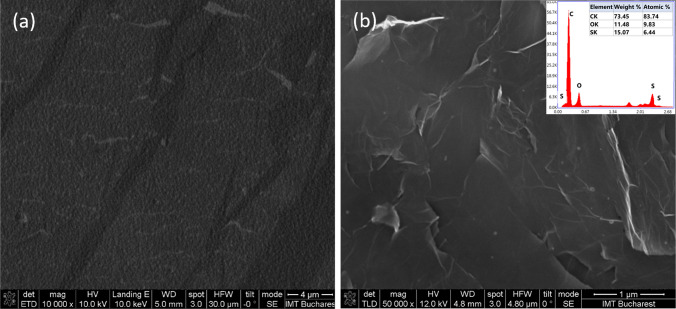
SEM images of (**a**) Bl-Gr/Au after the transfer process and (**b**) S-Gr deposited by drop casting on the Au sensing area of the sensor

In order to characterize the surface morphology and composition of the sensing materials (Bl-Gr/Au and S-Gr), SEM and EDAX techniques were used. The SEM micrographs of Bl-Gr/Au (Fig. 3(a)), after the transfer process illustrate the conformal deposition with minimal defects or discontinuities of the Bl-Gr on Au sensing area. Figure 3(b) presents the sulphur nanoparticles (S-NPs) distribution on the graphene sheet. The nearly spherically shaped S-NPs with a mean size of 24.7 ± 8.6 nm appear as individual entities, forming a scattered pattern across the graphene surface.

To further identify and quantify the elemental composition of the S-Gr material, energy-dispersive X-ray spectroscopy (EDX) was performed (inset Fig. 3(b)), highlighting the presence and composition of oxygen (O), carbon (C), and sulphur (S) in the sensing material.

**Fig. 4:**
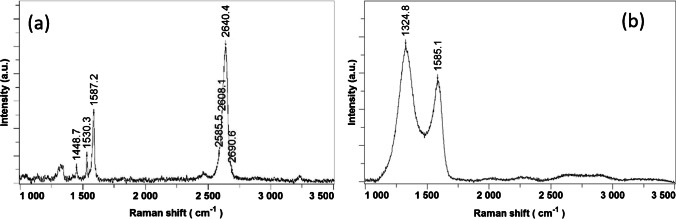
Raman spectra of (**a**) Bl-Gr/Au after the transfer process and (**b**) S-Gr/Au

A Raman spectroscope was further employed to analyze the molecular structure and lattice defects of the bilayer graphene transferred on the Au sensing area. The results presented in Fig. 4(a) highlight the corresponding graphene bands, G (1587.2 cm^−1^) and 2D, representing the sp^2^-bonded graphitic carbon atom and the double resonance of second-order processes involving two phonons, respectively. Some Raman bands corresponding to PMMA can also be observed at 1448.7 cm^−1^ and 1530.3 cm^−1^, which most likely appeared during the graphene transfer process, where PMMA was used as a sacrificial layer. The formation of additional bonds between graphene and remnants of PMMA could be a possible explanation for the width of the D band (~ 1330 cm^−1^), associated with the lattice defects. An important characteristic that can also be observed in Fig. 4(a) consists of four components of the 2D band, associated with the bilayer graphene. The full-width half maximum of 2640.4 cm^−1^ Raman band is 49.91 cm^−1^, which is much larger than single-layer graphene (~ 30 cm^−1^), which proves that the transfer process of the bilayer graphene has been successfully carried out. Moreover, the quality ratio I_2D_/I_G_ value is 1.97, corresponding to bilayer graphene [37].

From the I_D_/I_G_ ratio, where the D band is proportional to the number of point defects in the graphene material, the defect density can be estimated from the following formula [38]:

1 $${n}_{d}^{2} \left({cm}^{-2}\right)= \frac{5.9 \times {10}^{14}}{{E}_{L}^{4}}{\left[\frac{I(D)}{I(G)}\right]}^{-1}$$

where EL is the laser excitation energy in eV, ID_,_ and IG are intensities of the D and G band, respectively.

A value of 7.2 × 10^13^ cm^−2^ was obtained for the defect density in Bl-Gr material.

Figure 4 (b) presents the Raman spectrum acquired for the S-Gr sample. The D (1324.8 cm^−1^) and G (1585.1 cm^−1^) Raman bands, corresponding to graphene derivatives, were approximated by Gauss functions. No bands indicating the formation of C-S bonds were found [39]. The Raman spectrum of S-Gr is quite similar to graphene oxide, most likely due to the 7.5% oxygen content and low content of sulphur (2.0%—4.0%) in the material. The I_D_/I_G_ defect ratio has values within the 1.34—1.67 range, which indicates the existence of graphene oxide, having various degrees of reduction in the deposited layer.

The respective I_D_/I_G_ ratio in S-Gr material corresponds to a defect density of around 1.3 × 10^13^ cm^−2^, a value lower than that found in Bl-Gr material. The higher defect density in Bl-Gr material can indicate more adsorption sites for gas molecules, thus an increase in the sensing properties for the SAW sensor. Moreover, the XRD analysis of the S-Gr material supports the presence of S-doped graphene, reduced graphene oxide, and sulphur phases (Figure S4). The dislocation density for the graphene phase, indicative of the number of defects in the material, was around 8.8 × 10^12^ cm^−2^, which is close to the value resulted from Raman analysis.

### Gas sensing mechanism of the Gr-based SAW sensors

**Fig. 5:**
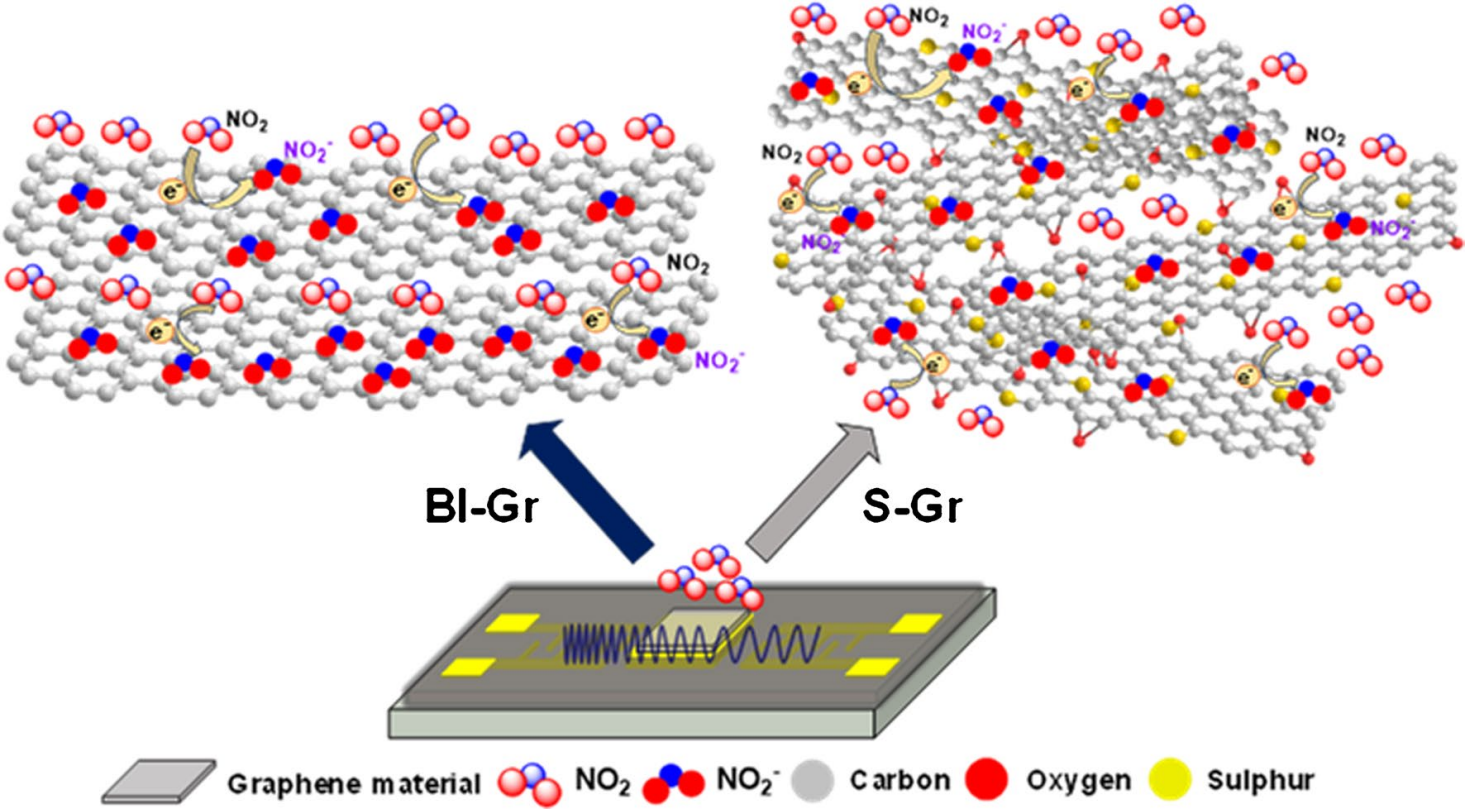
The schematic representation of the interaction mechanism between Gr-based nanomaterials and NO_2_ molecules

The interaction between the graphene-based sensing materials and the target gas is based on the chemisorption of the NO_2_ molecules on the graphene surface via the direct charge transfer process. The adsorption of target gas molecules on the sensing surface is due to the electron-withdrawing property of the NO_2_ molecule, which interacts with the electron-rich graphene surface, resulting in the NO_2_^−^ ions formation after the charge transfer (Fig. 5).

The surface changes occurring at the graphene interface with the target gas after the chemisorption process can modify the wave propagation properties by mass loading, elastic and acoustoelectric effects of the sensing film, according to the following equation [14, 24]:

2 $$\frac{\Delta f}{{f}_{0}}\cong \frac{\Delta v}{{v}_{0}}=-{C}_{m}{f}_{0}\Delta \left({\rho }_{s}\right)+4{C}_{e}{f}_{0}\Delta (h{G}{'})-\frac{{k}^{2}}{2}\Delta \left(\frac{1}{1+{\left(\frac{{v}_{o}{c}_{s}}{{\sigma }_{s}}\right)}^{2}}\right)$$

where $\Delta f$ and ${f}_{0}$ are frequency shift and the operating frequency; $\Delta v$ and ${v}_{0}$ represent the change in SAW velocity and unperturbed wave velocity; ${C}_{m}$ and ${C}_{e}$ are the sensitivity coefficients of the mass and elasticity; ${\rho }_{s}$ and $h$ are the surface mass density per unit area and the thickness of the sensing film; ${G}{'}$ and ${k}^{2}$ are the shear modulus and the electromechanical coupling coefficient; ${\sigma }_{s}$ and ${c}_{s}$ are the sheet conductivity of the sensing film and the capacitance per unit length of the device.

Since the developed graphene-based sensors use an Au sensing area between the IDTs (Fig. 1), as a substrate for the graphene-based sensing materials, the acousto-electric effect will no longer influence the resonance frequency of the sensors [24].

The Bl-Gr surface has a more available active area compared to monolayer graphene, thus enhancing the charge transfer towards NO_2_ molecules, and, at the same time, increasing the total number of absorbed molecules on the sensor’s sensing surface.

On the other hand, the sulphur atoms incorporated into the graphene lattice as a result of the replacement of carbon atoms during the synthesis process introduce defects into the graphene lattice, thus changing its electronic and chemical properties [40] and enhancing the adsorption of molecules onto the graphene surface [33].

### Electrical characterization of the Gr-based SAW sensors

#### Sensing performance in NO_2_ detection

To investigate the potential practical application of Bl-Gr and S-Gr towards NO_2_ sensing, the SAW sensors were further characterized in terms of LOD and selectivity. The sensors’ output signal was measured at room temperature (24 °C) in the presence of NO_2_ as the target gas, and formaldehyde (CH_2_O), ammonia (NH_3_), and carbon monoxide (CO) as possible interfering gases at 121 MHz operating frequency.

**Fig. 6:**
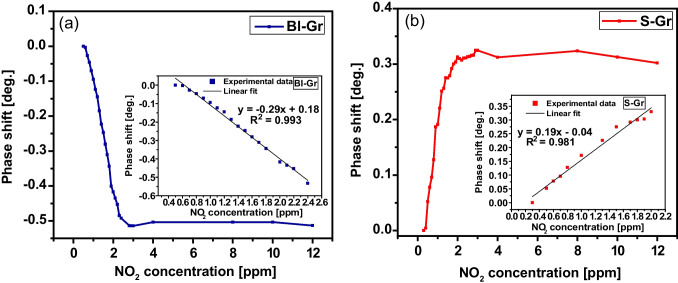
NO_2_ gas sensing response of the SAW sensors at 24 °C (insets: Phase shift plotted versus the NO_2_ concentration) using (**a**) Bl-Gr and (**b**) S-Gr sensing layers

The data reported in Fig. 6 were calculated as the difference in phase between the corresponding values measured in the 0.3—12 ppm range and the value recorded for the lowest gas concentration. Since our tests have shown that the sensor’s response reaches its steady-state value in less than 300 s after each increase in NO_2_ concentration in the measurement chamber, the time interval between two consecutive measurements has been set to 5 min.

As shown in the inset of Fig. 6(a), by plotting the Bl-Gr SAW sensor phase change as a function of NO_2_ gas concentration, a linear regression of data has been fitted in the 0.5—2.4 ppm measured concentration range, suggesting the advantage of using this sensor in low- NO_2_ concentration detection. A decrease of 0.53º in the sensor response for the 0.5—2.4 ppm concentration range was observed for the Bl-Gr SAW sensor. The sensitivity of the Bl-Gr SAW sensor toward NO_2_ has been measured by the slope of the calibration curve as 0.29º/ppm, a value comparable to other data reported in the literature [10]. The origin of this high sensitivity was attributed to the elastic effect (Eq. 2) of the graphene sensing material. From the linear regression of data depicted in Fig. 6(a) for the Bl-Gr SAW sensor, the LOD of 0.068 ppm, calculated as 3.3 σ/S (σ is the standard error of the linear regression slope and S is the slope of the calibration curve), has been achieved at room temperature.

In the same way, the variation of the phase shift with the NO_2_ gas concentration for the S-Gr SAW sensor was plotted in Fig. 6(b), highlighting a linear regression fit in the 0.3 – 2 ppm concentration range. In this case, an increase of 0.3º in the output signal phase was observed for the S-Gr functionalized SAW sensor, and a sensitivity of 0.19º/ppm was obtained from the slope of the calibration curve in the linear range. Moreover, an LOD of 0.140 ppm was obtained at RT for the S-Gr SAW sensor. The sensitivity of the S-Gr SAW-based sensor was attributed mainly to the mass loading effect, according to Eq. 2, since the phase shift increased with the NO_2_ concentration.

The LOD values of both SAW sensors, in the sub-ppm domain range, are lower than other reported values in previous literature, as summarized in Table S2, which includes SAW devices with different carbon-based sensing materials for NO_2_ detection, suggesting the high potential of the sensors for practical RT based gas sensing applications, especially of the as-prepared Bl-Gr/LiTaO_3_ sensor.

The sensing performances of the Bl-Gr SAW sensor were superior in terms of sensitivity, LOD, and linear range domain as compared to the S-Gr functionalized sensor (Table 1). Although the S-Gr SAW sensor showed lower sensing performances, it has a great advantage in the functionalization process for mass production of the NO_2_ sensors with good reproducibility, involving only two simple steps (Figure S3). On the other hand, the functionalization protocol using Bl-Gr is a more challenging process, carried out by successive depositions of a single layer of graphene, in which each step can create defects in the graphene structure, which subsequently affects the sensing performance of the NO_2_ SAW sensor.

**Table 1:** Analytical parameters of the Gr-based SAW sensors measured for NO_2_

Analytical parameters	Bl-Gr	S-Gr
Linear range (ppm)	[0.5–2.4]	[0.3–2]
Slope (deg./ppm)	-0.291	0.190
Standard error of the slope	0.00605	0.00802
Intercept (deg.)	0.183	-0.035
Correlation coefficient	0.993	0.981
LOD (ppm)	0.068	0.140
LOQ* (ppm)	0.208	0.423

*Limit of quantification LOQ = *10σ/S, where σ is the standard error of the linear regression slope and S is the slope of the calibration curve*

#### Selectivity of the Gr-based SAW sensors

**Fig. 7:**
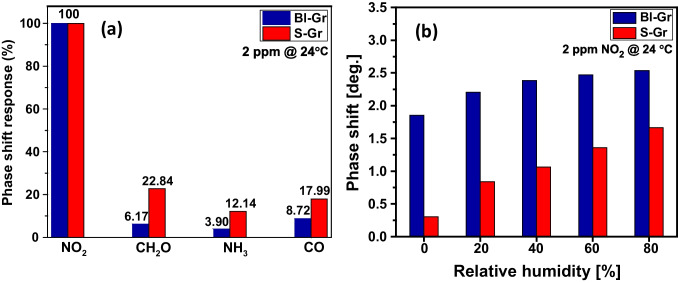
(**a**) Selectivity test of Bl-Gr (blue) and S-Gr (red) based sensors towards various interfering gases at RT; (**b**) NO_2_ sensing performances of Bl-Gr (blue) and S-Gr (red) based sensors under different relative humidity condition at RT

To evaluate the selectivity of the developed SAW-based sensors, gas sensing tests were performed at RT towards various possible interfering gas species, such as CH_2_O, NH_3_, and CO. In Fig. 7(a), the phase shift responses for both Bl-Gr and S-Gr-based sensors for each tested gas species were evidenced, where the response in percentile is defined as the ratio between the phase shift of the direct transfer coefficient S_21_ resulting from the sensor in the presence of 2 ppm interfering gas, and the phase shift of the sensor in the presence of 2 ppm NO_2_.

It can be observed that both sensors exhibit high phase shift responses for the NO_2_ gas compared to the other interfering gas species. Notably, the recorded response of the Bl-Gr SAW sensor showed small changes in phase shift for the interfering gases, showcasing the high selectivity of the developed sensor to detect NO_2_ at room temperature with no influence from other gas molecules, with negligible shift response. However, the S-Gr SAW sensor displayed significant changes in a phase shift in the presence of some interfering gases, especially for CH_2_O, evidencing a lower selectivity towards NO_2_.

The SAW responses indicate that the Bl-Gr nanomaterial is more suitable for NO_2_ detection, outlining its great potential as a sensing coating for the development of NO_2_-SAW sensors. Moreover, as compared to the S-Gr SAW sensor, the Bl-Gr-based sensor presented better performance also in terms of linear range, sensitivity, and LOD (Table 1).

#### The sensors’ response in relative humidity conditions and stability

To further evaluate the influence of relative humidity (RH) on gas sensors’ performance, measurements were carried out at 2 ppm NO_2_ concentration under various RH values, ranging between 20 and 80% at RT, as depicted in Fig. 7(b).

It can be noticed that the Bl-Gr-based sensor shows a larger phase shift variation at 0% RH compared to the S-Gr sensor, presenting thus an enhanced sensitivity towards NO_2_ gas. At increasing RH levels, it was observed a gradual increase in the phase shift signal for both sensors, however, the variation of phase shift signal recorded for the Bl-Gr sensor was notably lower, whereas the S-Gr sensor exhibits a five-fold increase in phase shift at the highest RH value of 80%. These results indicate that the Bl-Gr material has higher stability in humidity conditions, in comparison to the S-Gr material, emphasizing its great potential as a sensing coating for NO_2_ detection even in high RH levels.

The reproducibility of both SAW sensors was evaluated by measurements in the presence of 2 ppm NO_2_ at room temperature, showing good reproducibility, with no significant changes in phase shift responses (Figure S5a). The recovery of graphene-based SAW sensors was performed at room temperature by purging the gas chamber with nitrogen, for almost 2 h. The results showed that the recovery process is slow at room temperature, most probably due to the strong binding between NO_2_ and the sp^2^ carbons of graphene [41]. During the long-term stability measurements at 2 ppm NO_2_, considerable variations in the phase shift response were observed for both graphene-based sensors after a day between tests (Figure S5b), owing to the chemisorption of the NO_2_ molecules on the carbonic surface, which are slowly desorbed. Partial recovery of the response over time for both carbon-based sensors can be evidenced after several days, however, the S-Gr-based sensor showed a lower recovery rate in the response, due to the presence of oxygen species on the material surface which bind stronger to the NO_2_ molecules. This limitation has lately been addressed in several papers, and the main proposed approaches were UV light activation [42] and MXene graphene activation [43]. However, significant improvement in the recovery time can be also obtained by running successive thermal treatments at high temperatures to facilitate the gas desorption before other gas sensing experiments [44].

## Conclusion

DL-SAW devices with SiO_2_ guiding layer were designed and fabricated by surface micromachining on LiTaO_3_-36ºYX piezoelectric substrate. Bl-Gr and S-Gr sensing nanomaterials have been successfully deposited on as-fabricated SAW devices, via electrochemical delamination and drop-casting approaches, and demonstrated their potential applicability for the high-performance room temperature NO_2_ gas sensors. The sensitivity of Bl-Gr SAW sensors towards NO_2_, measured at room temperature, exhibited a value of 0.2º/ppm and an LOD of 0.068 ppm, whereas the S-Gr SAW sensor achieved a sensitivity of 0.19º/ppm and a LOD of 0.140 ppm. Although there are no significant differences regarding the sensitivity and LODs of the two sensors, the phase shift response showed much better selectivity for the Bl-Gr SAW sensors. Moreover, the Bl-Gr SAW sensor presented higher stability in humidity conditions, highlighting the great potential of Bl-Gr as a sensing coating for NO_2_ detection even in high RH levels. Due to its better performances (in terms of linear range, sensibility, LOD, and selectivity) as compared to the S-Gr SAW sensor, the Bl-Gr SAW sensor proves to be a very suitable option in wireless NO_2_ sensing platforms. Bl-Gr SAW sensor achieved a low LOD (68 ppb), which is one of the lowest values reported so far in literature for the SAW sensors, which use the phase variation of the transfer coefficient for NO_2_ detection. The origin of high sensitivity was attributed to the elastic effect of the Bl-Gr sensing material. Besides, the large specific surface area of the Bl-Gr enables the charge transfer towards NO_2_ molecules, increasing the total number of absorbed molecules on the sensor’s sensing surface. The Bl-Gr SAW sensor showed remarkable results, outlining its potential for sensitive and selective detection of NO_2_ at room temperature.

## Supplementary Information

Below is the link to the electronic supplementary material.

Supplementary file1 (DOCX 506 KB)

## Data Availability

The datasets generated during and/or analyzed during the current study are available from the corresponding authors on reasonable request.
