# Supplementary material for: The development of sensitive graphene-based surface acoustic wave sensors for NO2 detection at room temperature
Source: Mikrochim Acta. 2024 May 10;191(6):323. doi: 10.1007/s00604-024-06397-y (PMC11599405; doi:10.1007/s00604-024-06397-y)
Supplement: Supplementary file 1 — Supplementary file1 (DOCX 506 KB) [file 604_2024_6397_MOESM1_ESM.docx]

**Electronic Supplementary Material**

**The development of sensitive graphene-based surface acoustic wave sensors for NO_2_ detection at room temperature**

Valentin Buiculescu^˧^, Livia Alexandra Dinu^˧^, Lucia Monica Veca, Cătălin Pârvulescu, Madalina Mihai, Oana Brîncoveanu, Florin Comănescu, Costin Brașoveanu, Marius Stoian, Angela Mihaela Baracu^*^

National Institute for Research and Development in Microtechnologies (IMT Bucharest), 126A Erou Iancu Nicolae Street, 077190 Voluntari (Ilfov), Romania

*Corresponding author: [angela.baracu@imt.ro](mailto:angela.baracu@imt.ro)

˧ These authors contributed equally to this work

Table S1. Layout features of the SAW-DL devices [1].

| **Design parameters of the SAW-DL devices** | |
| --- | --- |
| Wavelength: λ | 34 µm |
| Electrode width: λ/4 | 8.5 µm |
| IDT aperture: 70·λ | 2380 µm |
| Spacing between IDTs: 100·λ | 3400 µm |
| Spacing between IDTs and sensing area | 190 µm |
| Sensing area | 9 × 10 ^6^ µm^2^ |


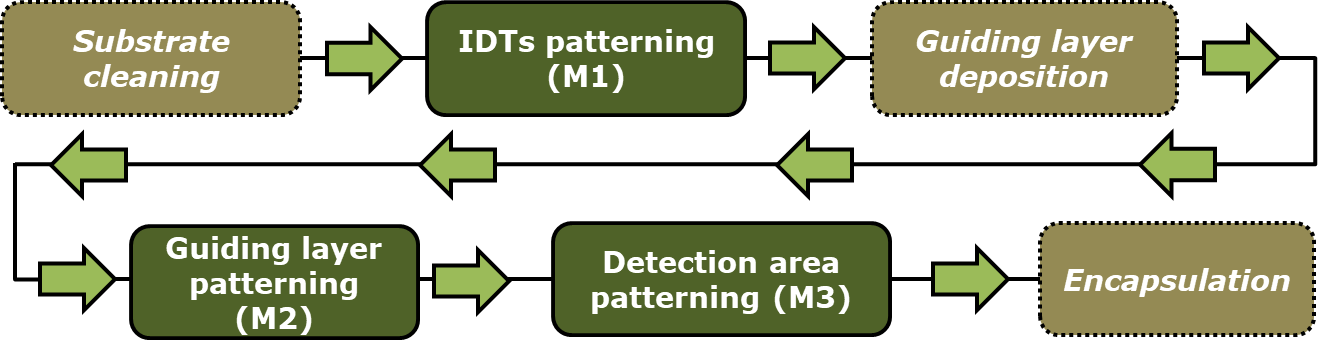


Figure S1. The technological flow of the SAW devices.

Open packages (OPs) made on FR-4 substrate as printed circuit boards (PCBs) with two metal layers (Figure S2) were used for chips’ encapsulation.

The geometry and dimensions of these OPs ensure their full compatibility with previously used packages [2, 3], made of low thermal expansion alloys and provided with glass feedthrough terminals.

Figure S2. Top and bottom views of the open package (OP).

*Legend: PTH - plated-through hole; PGW - pad for gold wire assembly; GCM - ground connection mark*

Each OP has four through-hole pads (PTH) on each Cu layer for the assembly of metal terminals using low melting temperature alloy bonding. Additionally, there are four pads (PGW) on the top layer, for thermal Au wire bonding to each signal pad of the SAW sensor. A Cu path defined in the top layer connects each PTH to the nearest PGW. The OP terminals used as ground connections for the input and output signal ports are positioned on the same side, marked as GCM in Figure S2.

|  |
| --- |

Figure S3. The functionalization protocol of SAW device using the S-Gr-based nanomaterial.

Powder X-ray diffraction (XRD) patterns were acquired with a 9 kW Rigaku SmartLab diffraction system with rotating anode, equipped with a CuK_α1_ radiation (λ = 1.5406 Å), operated at 45 kV and 100 mA, with *θ*/2*θ* from 10° to 70°, at a scanning rate of 4° min^–1^ and a 0.01° step width. Crystalline phases were identified using standard International Centre for Data Diffraction (ICDD) files and the average crystallite size of the phases was calculated by using the Scherrer equation:

$$D= \frac{K\lambda}{\beta cos\theta}$$

*where D is the mean crystallite size, K is the shape factor of the crystallites, taken as 0.93, λ is the Cu K_α1_ X-ray wavelength (1.5406 Å), θ is the Bragg diffraction angle, and β is the full width at half maximum (FWHM) of the diffraction line, in radians.*

The interplanar distance for the carbonic phases was calculated using Bragg’s law: *n*λ = 2*d*sinθ,$n\lambda=2d\cdot sin\theta$ $n\lambda=2d\cdot sin\theta$ where *n* is the diffraction order, *λ* is the X-ray wavelength, *d* is the interplanar distance, and *θ* is the Bragg diffraction angle.

The XRD pattern of the S-doped Gr material used on the SAW sensor revealed two intense peaks at 2θ = 24.40° and 25.92°, corresponding to a rGO phase and a multi-layer graphene (MLG) phase, respectively (Figure S4) [4, 5]. Using Bragg’s law, the interlayer distance for rGO was evaluated to be 0.365 nm, which was higher when compared to the interlayer distance of 0.337 nm found in graphite, due to the presence of oxygen functional groups [6]. The interlayer distance between the graphene-like layers in the MLG phase was also calculated, obtaining an interlayer distance of 0.344 nm, close to a crystalline graphite phase. This indicates an increase in interlayer spacing in the MLG phase, owing to the lattice doping with larger sulphur atoms. The lattice strain in the MLG phase was obtained from the following equation: $\varepsilon=(d_{MLG}-d_{graphite})/d_{graphite}$, resulting a lattice strain of around 2%. In addition, the broadness of the peak in XRD profile can be attributed to the presence of α-S phase with diffraction peaks at 2θ = 28.80°, 31.67°, 34.25° and 36.14°, which correspond to the (313), (044), (137) and (244) reflection planes, respectively, as identified for standard phase of orthorhombic sulphur (ICDD card no. 08-0247) [7]. For the phases found in the S-Gr material, the crystallite size was evaluated at around 8.6 nm for the rGO phase, 3.4 nm for the MLG phase and 3.7 nm for the sulphur phase. The dislocation density (δ), indicative of the number of defects in the material, can be calculated using the following formula: δ = 1/*D*^2^, where *D* is the crystallite size of the phase [8]. A dislocation density for the graphene phase in the S-Gr material of around 8.8 × 10^12^ cm^-2^ was obtained, a value close to the one resulting from Raman analysis.

Figure S4. The XRD pattern of the S-Gr material.

**(b)**

**(a)**

Figure S5. (a) Reproducibility of Bl-Gr (blue) and S-Gr (red) based sensors in presence of 2 ppm NO_2_ at RT; (b) Response stability in time of Bl-Gr (blue) and S-Gr (red) based sensors.

Table S2. NO_2_ gas sensing properties of different graphene SAW-based sensors reported in previous literature.

| No. | Substrate | Sensing material | Target gas | Limit of detection (ppb) | Sensitivity | Sensor configuration | Operating temperature (ᵒC) | Linear range  (ppm) | Reference |
| --- | --- | --- | --- | --- | --- | --- | --- | --- | --- |
| 1 | 36ᵒ YX LiTaO3 | SWCNTs-CdA | NO_2_ | 160 | 0.3ᵒ/ppm | SAWR | RT | 2-10 | [9] |
| 2 | ST-Quartz | Graphene ink | NO_2_ | 300 | 25 Hz/ppm | SAWR-based dual oscillator | RT | 0.2-3 | [10] |
| 3 | Y-cut 128ᵒ LiNbO_3_ | PPy-rGO /Ag | NO_2_ | 2370 | 127.68 Hz/ppm | SDL | RT | 10-100 | [11] |
| 4 | Langasite | GO-PEDOT-PSS | NO_2_ | 175 | 57 Hz/ppm | SAWR | RT | 5-100 | [12] |
| 5 | Langasite | B@GCN QDs | NO_2_ | 7.9 | 35.9 kHz/50ppm | SAWR | RT | 0.5-50 | [13] |
| 6 | Langasite | GCN NRs | NO_2_ | 42 | 2.3 kHz/ppm | SDL | RT | 0.5-10 | [14] |
| 7 | ST-Quartz | MXene/GO | NO_2_ | - | 260 Hz/ ppm | SAWR | RT | 1-200 | [15] |
| 8 | 36ᵒ YX LiTaO3 | Bl-Gr | NO_2_ | 68 | 0.3ᵒ/ppm | SDL | RT | 0.5-2.4 | This work |
| 9 | 36ᵒ YX LiTaO3 | S-Gr | NO_2_ | 140 | 0.2ᵒ/ppm | SDL | RT | 0.3-2 | This work |

Legend: SAWR – SAW resonator; RT – room temperature; SWCNTs- single-walled carbon nanotubes; CdA – cadmium arachidate; PPY - polypyrrole; rGO - reduced graphene oxide; SDL – SAW delay line; GO - graphene oxide; PEDOT – Poly(3,4-ethylenedioxythiophene); PSS- polystyrene sulfonate; B@GCN QDs -Boron doped graphitic carbon nitride; GCN NRs - graphitic carbon nitride nanoribbons; MXene/GO - MXene-activated GO.

**References**

1. Baracu AM, Buiculescu V, Dinu LA, et al (2022) Surface acoustic wave sensors for NO2 detection based on sulfur-doped graphene. In: 2022 International Semiconductor Conference (CAS). IEEE, pp 269–272

2. Baracu A, Gurban A-M, Giangu I, et al (2015) Selective chemical sensor for liquid specimens based on lithium tantalate surface acoustic wave devices. In: 2015 International Semiconductor Conference (CAS). IEEE, pp 271–274

3. Puiu M, Zamfir L-G, Buiculescu V, et al (2018) Significance Testing and Multivariate Analysis of Datasets from Surface Plasmon Resonance and Surface Acoustic Wave Biosensors: Prediction and Assay Validation for Surface Binding of Large Analytes. Sensors 18:3541. https://doi.org/10.3390/s18103541

4. Dinu Gugoasa LA, Pogacean F, Kurbanoglu S, et al (2021) Graphene-Gold Nanoparticles Nanozyme-Based Electrochemical Sensor with Enhanced Laccase-Like Activity for Determination of Phenolic Substrates. J Electrochem Soc 168:067523. https://doi.org/10.1149/1945-7111/ac0c32

5. Golsheikh AM, Huang NM, Lim HN, Zakaria R (2014) One-pot sonochemical synthesis of reduced graphene oxide uniformly decorated with ultrafine silver nanoparticles for non-enzymatic detection of H2O2 and optical detection of mercury ions. RSC Adv 4:36401–36411. https://doi.org/10.1039/c4ra05998k

6. Gupta B, Kumar N, Panda K, et al (2017) Role of oxygen functional groups in reduced graphene oxide for lubrication. Sci Rep 7. https://doi.org/10.1038/srep45030

7. Devi LG, Arunakumari ML (2014) Synergistic effect between orthorhombic α-Sulfur and TiO2 as co-photocatalysts for efficient degradation of methylene blue: A mechanistic approach. J Mol Catal A Chem 391:99–104. https://doi.org/10.1016/j.molcata.2014.04.012

8. Khawal HA, Gawai UP, Asokan K, Dole BN (2016) Modified structural, surface morphological and optical studies of Li3+ swift heavy ion irradiation on zinc oxide nanoparticles. RSC Adv 6:49068–49075. https://doi.org/10.1039/c6ra04803j

9. PENZA M, AVERSA P, CASSANO G, et al (2007) Layered SAW gas sensor with single-walled carbon nanotube-based nanocomposite coating. Sens Actuators B Chem 127:168–178. https://doi.org/10.1016/j.snb.2007.07.028

10. Thomas S, Cole M, De Luca A, et al (2014) Graphene-coated Rayleigh SAW Resonators for NO2 Detection. Procedia Eng 87:999–1002. https://doi.org/10.1016/j.proeng.2014.11.328

11. Xiong S, Zhou J, Wu J, et al (2021) High Performance Acoustic Wave Nitrogen Dioxide Sensor with Ultraviolet Activated 3D Porous Architecture of Ag-Decorated Reduced Graphene Oxide and Polypyrrole Aerogel. ACS Appl Mater Interfaces 13:42094–42103. https://doi.org/10.1021/acsami.1c13309

12. Pasupuleti KS, Reddeppa M, Nam D-J, et al (2021) Boosting of NO2 gas sensing performances using GO-PEDOT:PSS nanocomposite chemical interface coated on langasite-based surface acoustic wave sensor. Sens Actuators B Chem 344:130267. https://doi.org/10.1016/j.snb.2021.130267

13. Pasupuleti KS, Ghosh S, Jayababu N, et al (2023) Boron doped g-C3N4 quantum dots based highly sensitive surface acoustic wave NO2 sensor with faster gas kinetics under UV light illumination. Sens Actuators B Chem 378:133140. https://doi.org/10.1016/j.snb.2022.133140

14. Pasupuleti KS, Vidyasagar D, Ambadi LN, et al (2023) UV light activated g-C3N4 nanoribbons coated surface acoustic wave sensor for high performance sub-ppb level NO2 detection at room temperature. Sens Actuators B Chem 394:134471. https://doi.org/10.1016/j.snb.2023.134471

15. Li X, Feng Y, Long J, et al (2024) MXene-activated graphene oxide enhancing NO2 capture and detection of surface acoustic wave sensors. Sens Actuators B Chem 401:135006. https://doi.org/10.1016/j.snb.2023.135006
